# Computational study of aortic hemodynamics for patients with an abnormal aortic valve: The importance of secondary flow at the ascending aorta inlet

**DOI:** 10.1063/1.5011960

**Published:** 2018-03-16

**Authors:** S. Pirola, O. A. Jarral, D. P. O'Regan, G. Asimakopoulos, J. R. Anderson, J. R. Pepper, T. Athanasiou, X. Y. Xu

**Affiliations:** 1 Department of Chemical Engineering, Imperial College London, South Kensington Campus, London SW7 2AZ, United Kingdom; 2 Department of Surgery and Cancer, Imperial College London, St. Mary's Hospital, London W2 1NY, United Kingdom; 3 Institute of Clinical Science, Imperial College London, Hammersmith Hospital, London W12 0HS, United Kingdom; 4 Royal Brompton and Harefield NHS Foundation Trust, Sydney Street, London SW3 6NP, United Kingdom; 5 Hammersmith Hospital, Imperial College Healthcare NHS Trust, Du Cane Road, London W12 0HS, United Kingdom

## Abstract

Blood flow in the aorta is helical, but most computational studies ignore the presence of secondary flow components at the ascending aorta (AAo) inlet. The aim of this study is to ascertain the importance of inlet boundary conditions (BCs) in computational analysis of flow patterns in the thoracic aorta based on patient-specific images, with a particular focus on patients with an abnormal aortic valve. Two cases were studied: one presenting a severe aortic valve stenosis and the other with a mechanical valve. For both aorta models, three inlet BCs were compared; these included the flat profile and 1D through-plane velocity and 3D phase-contrast magnetic resonance imaging derived velocity profiles, with the latter being used for benchmarking. Our results showed that peak and mean velocities at the proximal end of the ascending aorta were underestimated by up to 41% when the secondary flow components were neglected. The results for helical flow descriptors highlighted the strong influence of secondary velocities on the helical flow structure in the AAo. Differences in all wall shear stress (WSS)-derived indices were much more pronounced in the AAo and aortic arch (AA) than in the descending aorta (DAo). Overall, this study demonstrates that using 3D velocity profiles as inlet BC is essential for patient-specific analysis of hemodynamics and WSS in the AAo and AA in the presence of an abnormal aortic valve. However, predicted flow in the DAo is less sensitive to the secondary velocities imposed at the inlet; hence, the 1D through-plane profile could be a sufficient inlet BC for studies focusing on distal regions of the thoracic aorta.

## INTRODUCTION

Over the last few decades, evidence has been presented linking arterial hemodynamics to the initiation and development of cardiovascular diseases (Ku *et al.*, 1985; Caro *et al.*, 1971; Mohamied *et al.*, 2015; Friedman *et al.*, 1981; Zarins *et al.*, 1983; and Humphrey and Taylor, 2008). Particularly, it has been shown that wall shear stress (WSS) and bulk flow structures may have a strong influence on the formation of atherosclerotic plaques and aneurysms. Although WSS can be estimated from *in vivo* data acquired by phase-contrast magnetic resonance imaging (PC-MRI) (Piatti *et al.*, 2017a; 2017b), accuracy is still a major concern due to uncertainties in determining where the wall is and inadequate spatial resolution for near wall velocity measurements (Piatti *et al.*, 2017a). Computational modelling, on the other hand, has been widely employed and recognized as a preferred option for evaluating WSS when combined with patient-specific imaging. However, computational results can be strongly influenced by the employed boundary conditions (BCs) (Morbiducci *et al.*, 2013; Pirola *et al.*, 2017; Gallo *et al.*, 2012; and Caballero and Laín, 2013).

Advancements in imaging techniques have made it possible to acquire high resolution anatomical images and *in vivo* velocity measurements. The former can be processed to reconstruct patient-specific geometries, while the latter allows the extraction of realistic boundary conditions for computational fluid dynamics (CFD) simulations (Tan *et al.*, 2011; Cheng *et al.*, 2016; Pirola *et al.*, 2017; and Youssefi *et al.*, 2017). PC-MRI has been widely used to obtain velocity profiles which can be one-dimensional (1D, through-plane velocity component only) or three-dimensional (3D, through-plane and in-plane velocity components). However, the acquisition of 3D velocity components is not usually included in a standard imaging protocol for clinical examination. As a result, the through-plane velocity component is often acquired and used as inlet BC in patient-specific CFD studies (Youssefi *et al.*, 2017 and Tan *et al.*, 2011) instead of a full set of 3D velocities.

The difference between 1D and 3D velocity profiles as inlet BCs has been investigated by various authors. Goubergrits *et al.* (2013) compared the use of flat and MRI-based velocity profiles as inlet boundary conditions for CFD analysis of steady flow in aortic coarctation, showing that the evaluation of maximum WSS and pressure drop could be sensitive to secondary flow at the inlet plane. Van Doormaal *et al.* (2012) compared flat and MRI-based mouse-specific and non-specific velocity profiles for pulsatile flow in mouse aortic models, concluding that idealized velocity profiles should be avoided in studies focusing on the hemodynamics of the aortic arch (AA). The use of 3D time-varying velocity profiles was also assessed for CFD modelling of the carotid bifurcation (Campbell *et al.*, 2012), which showed that the bifurcation geometry had a stronger influence than inlet velocity profiles. A similar study of abdominal aortic aneurysms suggested that 1D velocity profiles would be sufficient for WSS evaluation, but 3D profiles must be used for the evaluation of helical flow features in abdominal aortic aneurysms (Hardman *et al.*, 2013).

The influence of inlet BCs on the evaluation of aortic hemodynamics was previously investigated by Morbiducci *et al.* (2013), who showed that 1D velocity profiles may be an adequate inlet BC for CFD to capture disturbed wall shear distributions in normal aortas. In healthy subjects, valvular velocity profiles are usually symmetric with a predominant axial component. However, this may not be true in patients with valve pathology or a prosthetic valve, which tends to cause highly skewed velocity profiles with significant secondary flow components. It is not clear to what extent the predicted flow field would be affected by the type of inlet BC for this cohort of patients. Therefore, this study aims to investigate and compare the influence of different inlet BCs on the results of CFD analysis of aortic hemodynamics in the presence of an abnormal aortic valve. For this purpose, two cases were analysed: one presenting a stenotic valve and the other with a mechanical valve (MV). Three inlet BCs were compared for each case: full 3D velocity profiles obtained from the patients' PC-MR images; 1D velocity profile (through-plane component, TP) derived from the corresponding 3D velocity vector; and flat velocity profile for the same flow waveform obtained from PC-MRI.

## RESULTS

### Velocity

Velocities at the model inlet were decomposed into in-plane and through-plane (or normal) components for visualisation and comparison. Table I compares the mean and maximum values of the velocity magnitude calculated from 3D velocity components with those calculated from the normal component alone for the two studied subjects. These results show that using the normal velocity component alone would underestimate the mean velocity by 41% [aortic valve stenosis (AVS)] and 33% (MV) and the maximum velocity by 19% (AVS) and 23% (MV).

**TABLE I.** Mean and maximum values of the velocity magnitude at the model inlet. These were calculated from 3D velocity components (Total) or just the normal component (Normal) for the two studied subjects (AVS, aortic valve stenosis; MV, mechanical valve).

	V_mean_ (range) (m s^−1^)		V_max_ (range) (m s^−1^)	
	Normal	Total	Normal	Total
AVS	0.16 (0.03–0.52)	0.27 (0.09–0.77)	0.66 (0.13–2.41)	0.82 (0.23–2.77)
MV	0.14 (0.04–0.47)	0.21 (0.07–0.67)	0.39 (0.12–1.26)	0.51 (0.16–1.72)

**FIG. 1.**
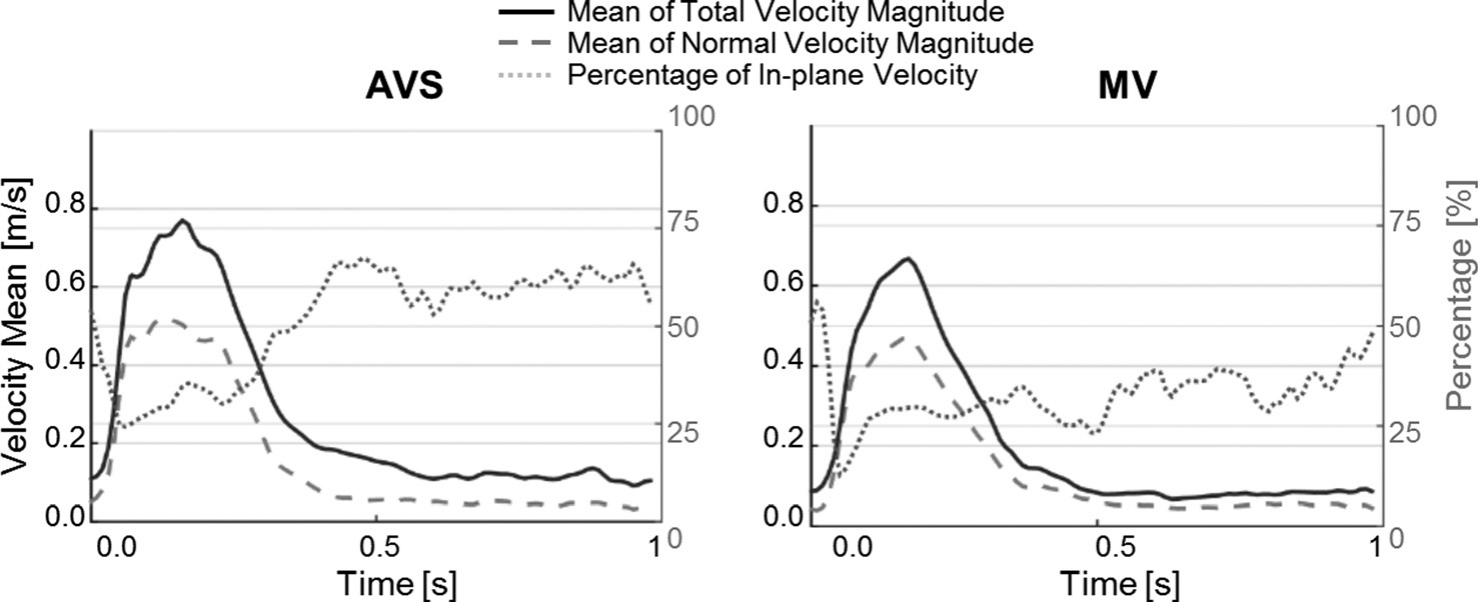
Variations of the total velocity magnitude (solid line), normal velocity magnitude (dashed), and ratio of in-plane to total velocities (dotted) over a cardiac cycle. Velocity values are in (m/s), and the ratio (right vertical axis) is expressed as a percentage.

Figure 1 shows variations over time of the total velocity and through-plane velocity magnitudes, along with the percentage of in-plane components with respect to the total velocity. On average, the in-plane velocity components constituted 51% (AVS) and 32% (MV) of the total velocity, with the maximum values being 67% (AVS) and 56% (MV) observed in diastole.

**FIG. 2.**
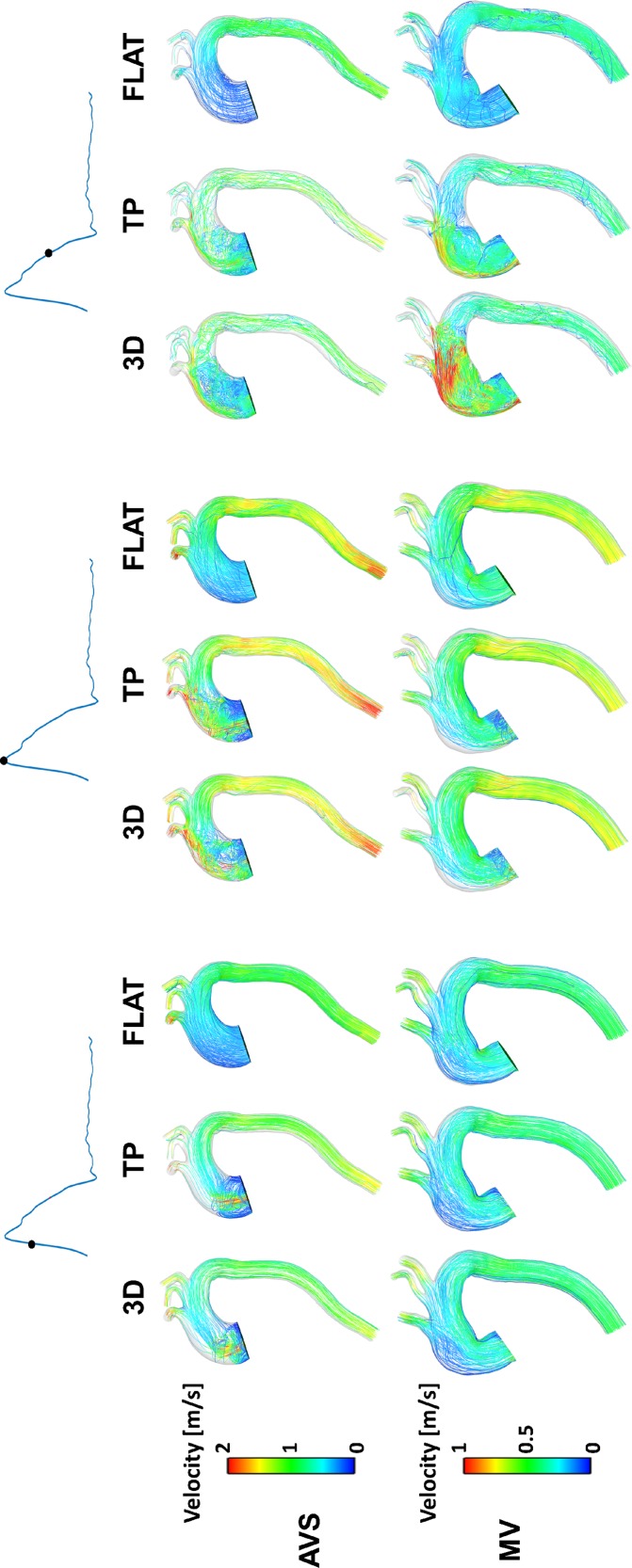
Velocity streamlines at three systolic time points.

From the CFD simulation results for both aorta models (AVS and MV), instantaneous velocity streamlines were captured at three systolic time points (Fig. 2). In general, flow patterns obtained with the 3D and TP inlet BCs were similar, while the FLAT BC produced different features especially in the ascending aorta (AAo) and aortic arch (AA). Flow patterns in the descending aorta (DAo) appeared to be less sensitive to the inlet BC applied. Comparison of the two aorta models showed a high velocity jet in the AAo of AVS during most of the systolic phase, which was not seen in MV until late systole.

**FIG. 3.**
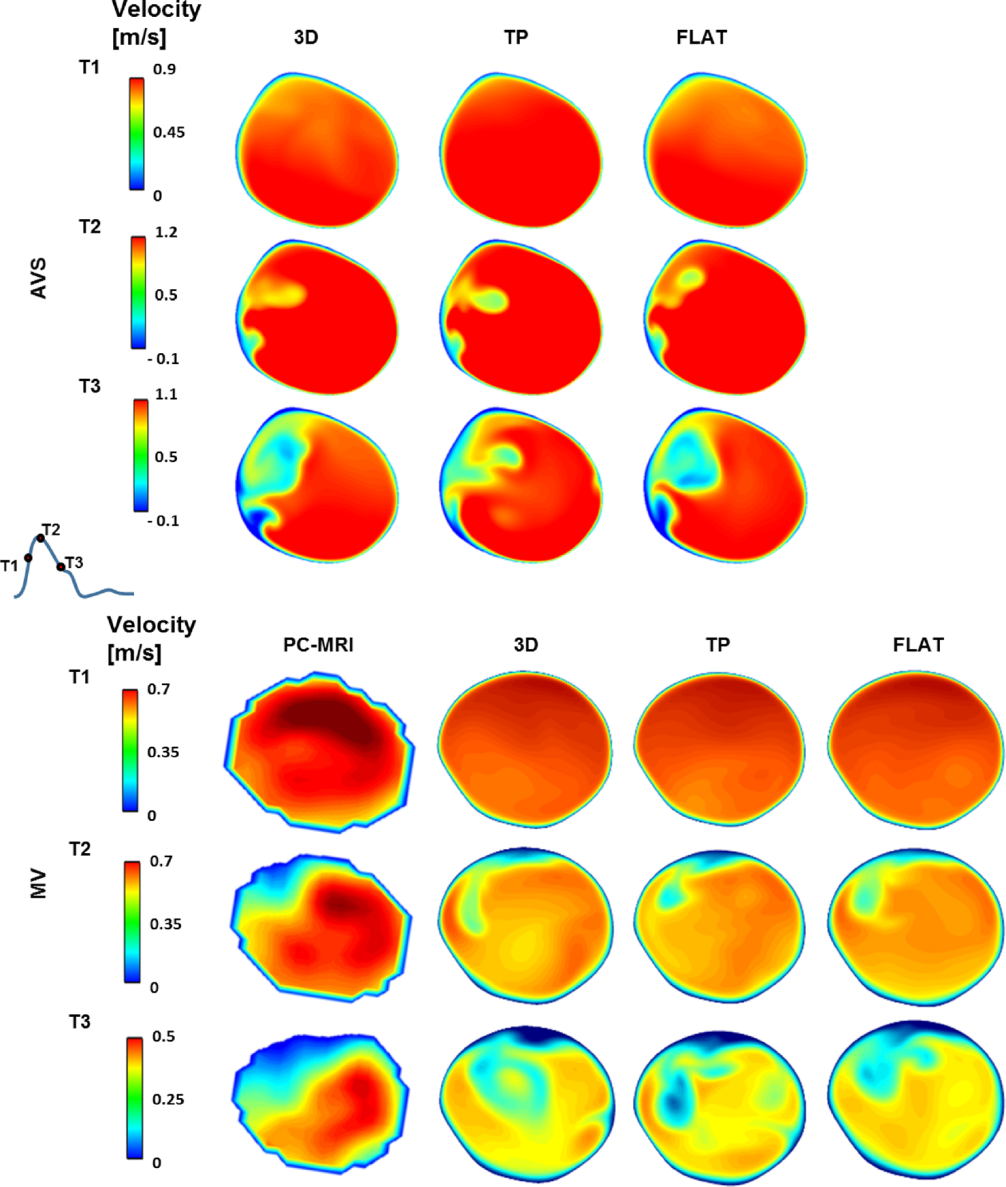
Axial velocity contours in the descending aorta at three systolic time points. Comparisons between computational results and PC-MRI data are presented for MV only, where CFD results are displayed at the same level of the PC-MRI plane. T1, mid-systolic acceleration; T2, peak systole; T3, mid-systolic deceleration. Positive values represent velocities in the head-foot direction.

Since PC-MRI was also acquired at a plane in the DAo, attempts were made to compare the CFD results with *in vivo* measurements at the same location. Figure 3 shows colour-coded axial velocity contours at the same level of the PC-MRI plane at three systolic time points for all simulated inlet BCs. Note that comparison with *in vivo* PC-MRI data was not possible for AVS since the acquired images showed the presence of a side branch from the aorta at the same level, making them unsuitable for direct comparison with the computational results (as the CFD model did not include this side branch). It can be seen that all three inlet BCs produced similar contour maps for both AVS and MV, and in the case of MV, there was a good qualitative agreement with the PC-MRI data.

### Helicity

**FIG. 4.**
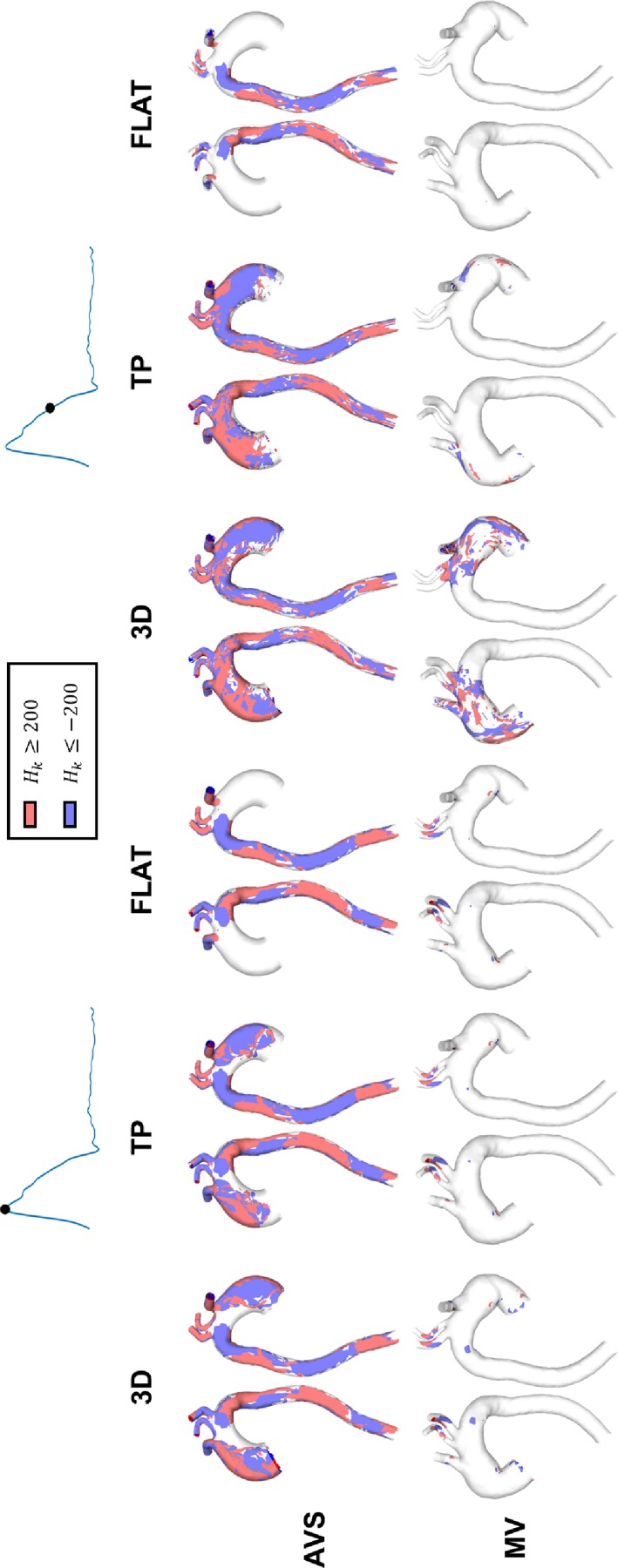
Isosurfaces of helicity density ($H_{k}$) for case AVS (first row) and case MV (second row), for the three studied inlet BCs. The isosurfaces were generated for $\left|H_{k}\right|\geq 200$ (red colour represents $H_{k}\geq 200$, and blue colour represents $H_{k}\leq -200$).

Figure 4 shows helicity density isosurfaces for both AVS and MV at peak systole and mid-systolic deceleration, where $\left|H_{k}\right|\geq 200$ corresponds to regions of high *H_k_* (Singh *et al.*, 2016a), with positive values of *H_k_* representing the clockwise helical flow and negative values representing the anti-clockwise helical flow.

It is clear that helicity was consistently higher in AVS than in MV, caused by the high velocity jet in the AAo and the sharp aortic curvature in AVS. Comparisons of results obtained with different inlet BCs suggested that for AVS, results with the 3D inlet BC showed higher values of helicity in the ascending aorta (AAo) and the arch at both time points although similar patterns were obtained with the TP inlet BC. The results with the FLAT BC showed significant differences, especially in the AAo and arch. For MV, small regions of high *H_k_* values were found at peak systole for all three inlet BCs; at mid-decelerating systole, the 3D BC produced more regions of high *H_k_* in the AAo and aortic arch, which were only partially captured by the TP BC but not with the FLAT BC. In the DAo of both AVS and MV, differences between results obtained with the three inlet BCs were negligible.

**FIG. 5.**
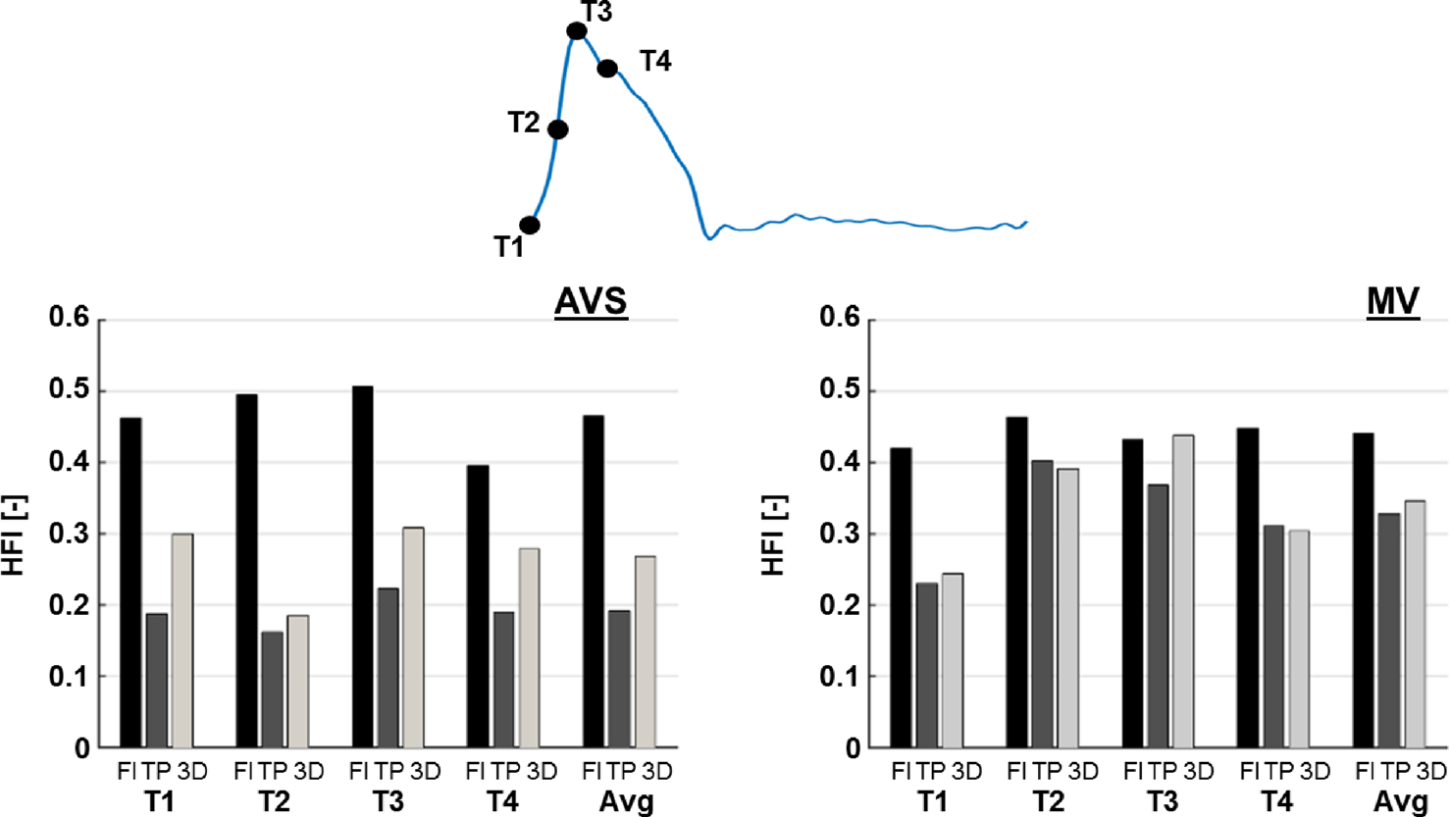
Comparison of HFI obtained with different inlet boundary conditions (FLAT = Fl; TP and 3D) for both AVS and MV. HFI was calculated with particles emitted at the four indicated systolic time points (T1–T4) and as average (Avg).

Figure 5 shows the helicity flow index (HFI) values obtained with particles emitted at the four selected systolic time points (T1–T4) and their average values, for both AVS and MV. FLAT BC significantly over-estimated HFI compared to the 3D BC, with the exception for T3 in MV. A maximum difference of +169.7% was observed at T2 for AVS, and differences in average HFI between the FLAT and 3D BCs were +73.6% and +28.0% for AVS and MV, respectively. On the other hand, HFI values in AVS were under-estimated with the TP inlet BC (maximum difference of −37.6% at T1 and −28.9% for average HFI), while in MV, differences between the TP and 3D results were relatively small (−4.9% for average HFI), with the exception for T3, where a difference of −16.1% was observed.

### Wall shear stress

**FIG. 6.**
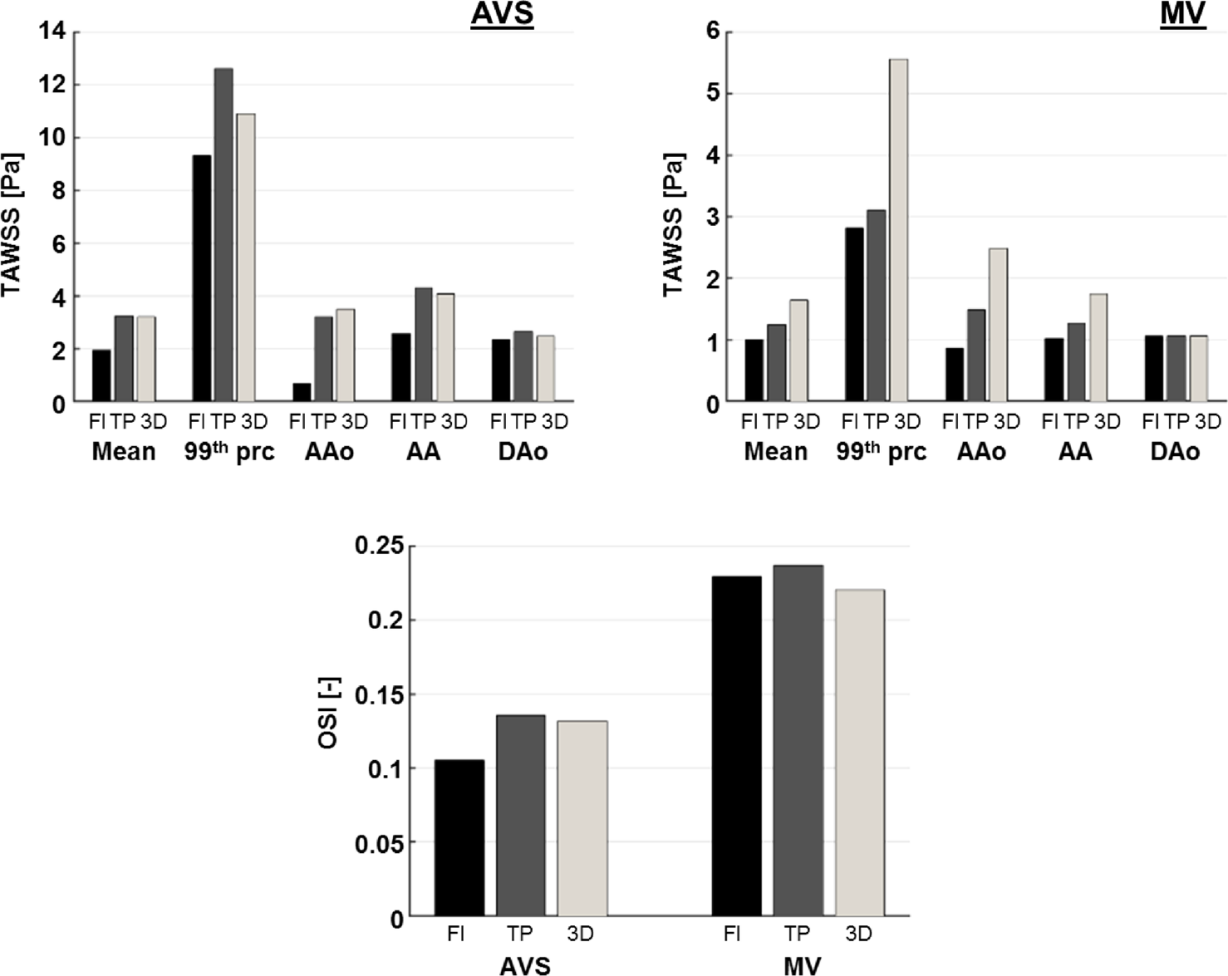
Comparison of TAWSS and OSI obtained with different inlet boundary conditions (FLAT = Fl; TP and 3 D) for both AVS and MV. For TAWSS [Pa], the following values are reported (left to right): surface mean value over the whole aorta (Mean), 99th percentile (99th prc), and surface mean value over the ascending aorta (AAo), the aortic arch (AA), and the descending aorta.

Figure 6 shows time-averaged WSS (TAWSS) and oscillatory shear index (OSI) values obtained for both cases with the three imposed inlet BCs. TAWSS values were compared for the surface mean over the entire aorta, maximum values (represented by the 99th percentile in order to exclude any spurious results at sharp corners), and surface mean values for each of the three main aortic sections: AAo, AA, and DAo. In AVS, the mean TAWSS was in good agreement between TP and 3D BCs, while FLAT BC underestimated the TAWSS mean value by 39.4% with respect to 3D BC. The 99th percentile value was underestimated by FLAT BC (−14.9%) and over-estimated by TP BC (+15.34%). In MV, both FLAT and TP BCs showed values lower than 3D BC, for both the mean and the 99th percentile: −39.3% for FLAT and −24.7% for TP and −49.4% for FLAT and −44.1% for TP, respectively.

However, larger differences were found with FLAT than with TP BC. For both AVS and MV, differences in TAWSS reduced along the aorta from the AAo to the DAo. In AVS, FLAT BC underestimated TAWSS values in the AAo (−80.2%) and AA (−37.3%), while TP showed an under-estimation in the AAo (−8.4%) and a slight overestimation in the AA (+4.9%). In MV, FLAT and TP BCs produced lower TAWSS than the reference case (3D) in both AAo (−65.1% and −40.1%, respectively) and AA (−41.4% and −27.2%, respectively). Overall, differences were much lower in the DAo, particularly for MV (−0.2% FLAT and +0.5% TP) compared to AVS (−4.5% and +7.3%).

Mean values for OSI were calculated over the whole aorta for both AVS and MV for the three inlet BCs (FLAT, TP, and 3D). TP BC slightly overestimated the OSI mean (+2.8% for AVS and +7.7% for MV), while FLAT showed lower values in AVS (−20.13%) and higher in MV (+4.0%).

**FIG. 7.**
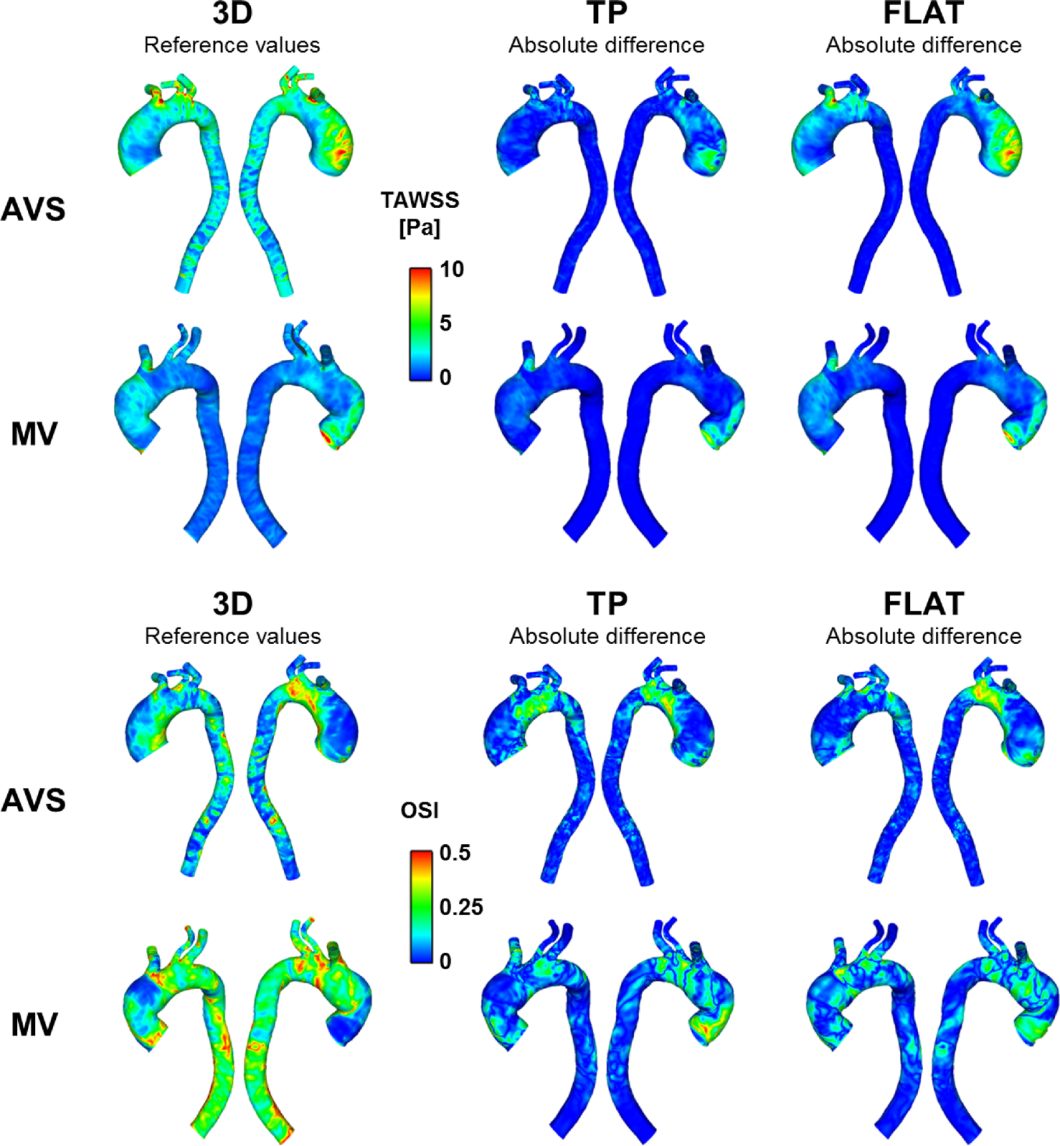
TAWSS [Pa] (first and second rows) and OSI (third and fourth rows) contours for the two cases (AVS and MV) obtained with 3D inlet BC (first column). These were used as reference values for the evaluation of absolute differences (second and third columns).

Figure 7 (first column) shows TAWSS and OSI results for AVS and MV obtained with the 3D inlet BC. These results were used as baseline values to calculate the absolute differences for TP and FLAT BCs, shown in Fig. 7 (second and third columns). For both TAWSS and OSI, differences were mainly located in the AAo, AA, and supra-aortic branch roots, while differences in the DAo were negligible. Furthermore, both qualitative and quantitative discrepancies were more pronounced with FLAT BC although large differences (>10 Pa for TAWSS and 0.5 for OSI) were found with both TP and FLAT.

**FIG. 8.**
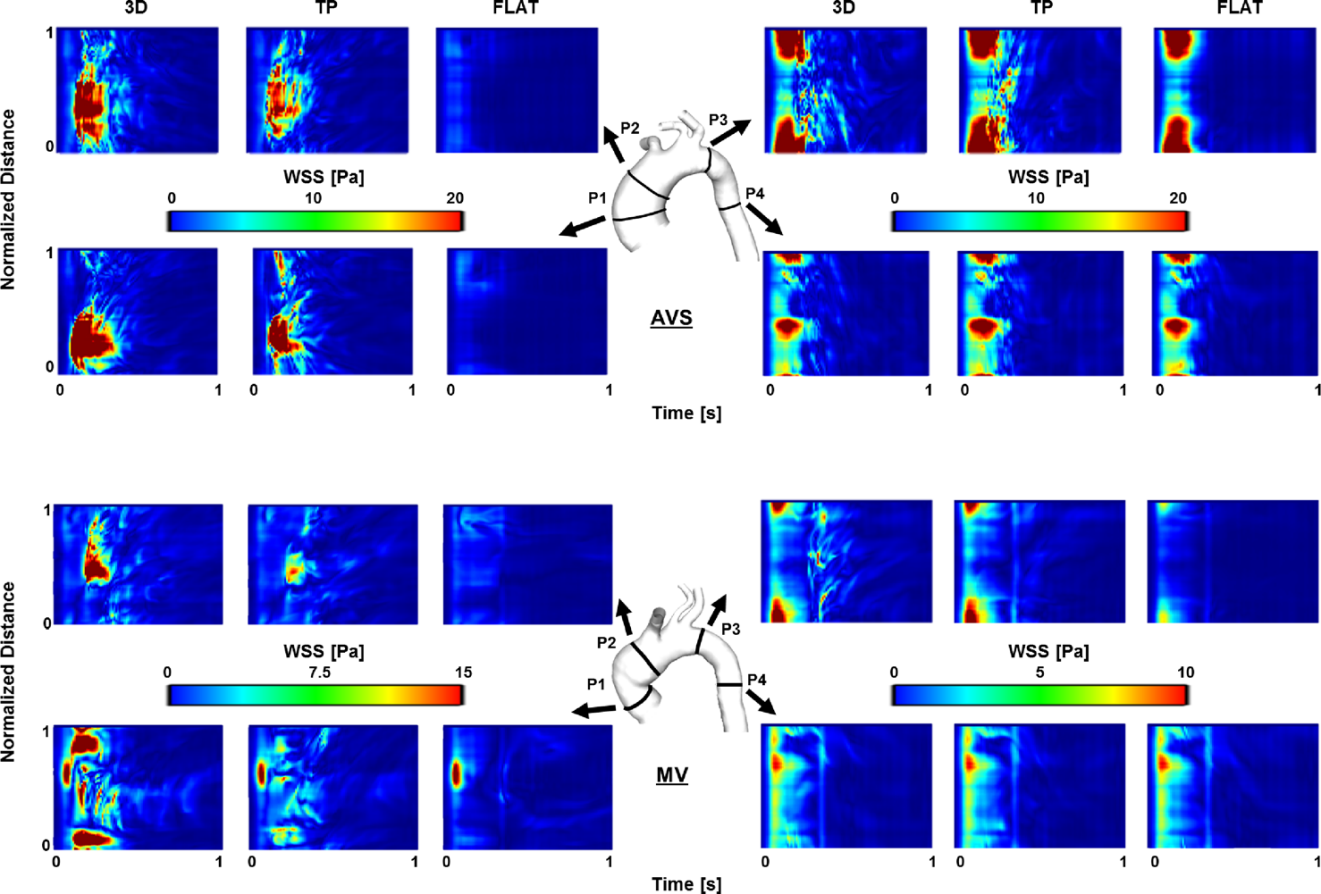
WSS (Pa) spatial and temporal maps for the two cases (AVS, top; MV, bottom). Maps were computed along four analysis planes, located in the ascending aorta, aortic arch, and proximal descending aorta (P1–P4, as shown). Planes are unwrapped along vertical axes, with distances along the circumference normalised for comparison purposes. The horizontal axes show the temporal evolution of the WSS values along the same plane.

To further examine the differences in WSS distribution, four transverse planes (P1–P4) were chosen in the two aorta models. Figure 8 shows comparisons of WSS temporal maps obtained with the three inlet BCs for AVS and MV. In general, all inlet BCs produced almost identical patterns at P4, but varying degrees of differences can be seen at other planes. The differences appeared to be more prominent in the AAo (P1 and P2) than in the distal arch (P3). At P1 and P2, TP BC produced patterns closer to those of 3D BC, but FLAT BC failed to capture the main features.

## DISCUSSION

Despite the fact that the role of hemodynamics in vascular health is well recognized (Friedman *et al.*, 1981; Ku *et al.*, 1985; and Mohamied *et al.*, 2015), direct *in vivo* quantification of key hemodynamic parameters (e.g., TAWSS and HFI) is not yet possible and their evaluation by PC-MRI is still limited by technological issues (Piatti *et al.*, 2017a). Therefore, image-based CFD has emerged as the method of choice in the study of aortic hemodynamics. However, as computational results depend strongly on the chosen boundary conditions (Pirola *et al.*, 2017; Morbiducci *et al.*, 2013; and Gallo *et al.*, 2012), rigorous assessments of all sources of uncertainties involved in the evaluation of hemodynamic parameters are needed.

A previous study by Morbiducci *et al.* (2013) investigated the effects of different inlet BCs on aortic hemodynamics in a healthy subject, showing that the TP BC may capture WSS and WSS-related indices (e.g., TAWSS) with sufficient accuracy. In the present study, we set out to investigate if this conclusion would hold for cases presenting an abnormal valve (i.e., pathological or mechanical valve). To this end, we studied the hemodynamics in a patient presenting a severe aortic valve stenosis (AVS) and a patient implanted with a mechanical valve (MV). The results obtained by imposing a flat profile (FLAT) or a 1D axial velocity component (TP) as inlet BCs were compared with those obtained by using 3D PC-MRI derived velocity profiles (3D). The latter were used for benchmarking.

Our results showed evidence of substantial in-plane velocity components (secondary flow) at the proximal end of the AAo in patients with an abnormal valve and that their effects on the helical flow structure in the aorta cannot be neglected. The velocity component analysis revealed that both mean and maximum values were significantly underestimated by using the normal velocity component alone. However, no qualitative differences in velocity contours were observed among the three different inlet BCs at the second PC-MRI acquisition plane located in the proximal DAo.

Furthermore, helical flow descriptors, such as helicity density (*H_k_*) and HFI (helicity flow index), were evaluated for quantitative comparison of bulk flow structures predicted with different BCs. Helical flow is generated by the rotation of blood about the axis along the main direction of flow. It is a common feature of normal and abnormal aortas, and helical flow has been shown to help to reduce flow stagnation and increase oxygen transport (Liu *et al.*, 2010 and Wen *et al.*, 2011). *H_k_* and HFI are two of the commonly used parameters to describe helical flow. *H_k_* is defined as the dot product of velocity and vorticity, whereas HFI is a Lagrangian-based descriptor which quantifies the degree of helicity using particle trace analysis and local normalized helicity (Morbiducci *et al.*, 2009). The obtained *H_k_* and HFI values for the two patients examined here were consistent with previous studies (Morbiducci *et al.*, 2007; Singh *et al.*, 2016a; and Morbiducci *et al.*, 2009), and our results demonstrated that the choice of inlet BC can have a strong influence on helicity evaluation in the AAo. Interestingly, TP inlet BC showed a tendency to underestimate HFI; this was particularly evident in the case presenting a valvular stenosis (AVS) where secondary flow was up to 67% of the total flow. As HFI describes the extent of helical flow (Morbiducci *et al.*, 2007), this finding suggests that in-plane velocity components in the aortic root have a strong influence on the formation of the helical flow structure in the aorta. On the other hand, FLAT BC appeared to overestimate HFI values, suggesting that the shape of the inlet velocity profile also plays an important role in determining the helical content of aortic flow.

WSS is also of interest since it has been associated with atheroprone regions, altered endothelial functions and vascular remodelling (Lee *et al.*, 2002; Liu *et al.*, 2003; and Wentzel *et al.*, 2005), possibly leading to severe aortic pathologies, such as dilatation, aneurysm, and dissection. For young healthy volunteers, TAWSS in the aorta was reported in the range of 0.25–0.33 Pa based on direct estimation from 4D flow MRI (Frydrychowicz *et al.*, 2009). WSS and its related parameters were determined using PC-MRI in patients with bicuspid aortic valves (BAV) by Barker *et al.* (2010), who found significant differences in systolic WSS values in the AAo between healthy and BAV subjects. They also reported higher shear circumferential asymmetry in BAV patients due to the eccentric systolic jet. In the present study, differences in WSS distribution were particularly marked in the ascending aorta, while these were negligible in the descending aorta. This finding can be inferred from the comparison of velocity contours shown in Fig. 3, demonstrating that hemodynamics in the distal AA and DAo is more influenced by the local geometry and the outlet BC (Pirola *et al.*, 2017) than the inlet BC imposed at the aortic root. Therefore, a flat velocity profile may be sufficient for studies which focus on hemodynamics of the distal aortic arch and descending aorta. In addition, OSI appears to be more sensitive to inlet BCs than TAWSS. As for the TAWSS magnitude, differences seem to be more pronounced on the right side of the ascending aorta. This is due to the presence of an eccentric velocity jet originating from the valve and impinging the AAo wall on its right side, a common feature in patients presenting abnormal valves (Entezari *et al.*, 2014 and Youssefi *et al.*, 2017). Overall, these results show that neglecting in-plane velocity components could possibly affect the identification of regions characterized by altered hemodynamics in the ascending aorta and aortic arch and, consequently, the understanding of disease development and progression.

Several assumptions were made in this study: distensibility of the aortic wall and transitional nature of the aortic flow were not included in the flow models. However, these assumptions do not invalidate the presented comparison, as the same assumptions were made in all cases. Furthermore, the effect of aortic root motion was not included. This has been shown to have a non-negligible impact on both aortic flow and wall stress (Jin *et al.*, 2003 and Singh *et al.*, 2016b). However, it may have had a greater influence on AVS than MV, as it has been shown that aortic root motion is significantly reduced in the presence of a mechanical valve, while it can be accentuated by aortic stenosis (Beller *et al.*, 2008). Unfortunately, information on aortic root motion was not available in the 2D PC-MRI data employed in this study.

Although comparison of CFD predictions with *in vivo* data was attempted, this was only possible for one acquisition plane in the descending aorta of one subject. Future studies will take advantage of more advanced imaging techniques, such as 4D flow MRI, for a more comprehensive comparison with *in vivo* data. The study could also be extended to evaluate the influence of inlet BC on pressure, as Goubergrits *et al.* (2013) showed that the presence of secondary flow at the model inlet could affect the estimation of pressure drop in aortic coarctation. Finally, a recent study by Bozzi *et al.* (2017) highlighted that some uncertainties involved in the evaluation of aortic hemodynamics may originate from issues related to the PC-MR imaging technique (e.g., signal to noise ratio). Such uncertainties would have been brought into the CFD analysis through the use of measured velocity profiles as inlet BCs. For example, the PC-MRI planes used to extract the inlet velocity profiles in this study had a thickness of 10 mm. This could compromise the accuracy of the derived velocities (especially the secondary velocity components) applied at the model inlet.

## CONCLUSIONS

The results presented in this study demonstrate the importance of including secondary flow components as part of the inlet BC for patient-specific evaluation of aortic hemodynamics and wall shear stress, especially for patients presenting an abnormal or pathological aortic valve. Based on these findings, it is recommended that 3D inlet velocity profiles should be used in future studies of hemodynamics in the ascending aorta and aortic arch. Although the results from the two cases examined here show that the 1D through-plane velocity profile could be a sufficient inlet BC for the evaluation of descending aortic flow and WSS, further case studies are needed to assess its impact especially for pathological cases involving diseases of the descending aorta.

## METHODS

MRI and PC-MRI of two subjects were acquired at the Robert Steiner MRI Unit (Hammersmith Hospital, London, UK) using an A 1.5 T Philips Achieva system (Best, Netherlands). The first patient (AVS) presented a bicuspid aortic valve (BAV) with severe aortic valve stenosis, while the second patient (MV) underwent aortic valve replacement with a mechanical valve (23 mm Sorin Carbomedics valve) and graft-conduit implantation (28 mm Maquet Hemashield Platinum graft) in the proximal section of the ascending aorta. Ethical approval for this study was granted by the Wales Research Ethics Committee in the United Kingdom (reference 14/WA/1225). Both patients gave informed consent before participating in this study.

Multi-slice sagittal anatomical images of the thoracic aorta and proximal vessels were acquired using a navigator-gated balanced steady-state free precession angiogram. PC-MR images were acquired at two planes perpendicular to the aortic axis: one was located above the aortic root and the other in the proximal descending aorta. Velocity was encoded along three main directions (foot-head, right-left, and anterior-posterior), with velocity encoding parameters (VENC) being set at 10% above the expected peak velocity for each component. Retrospective cardiac gating was employed in order to obtain 100 time points per average cardiac cycle. Voxel sizes were 0.5 × 0.5 × 2 mm and 1.4 × 1.4 × 10 mm for MRI and PC-MRI, respectively. Patients' pressure was measured 30 min prior to the MR scan by using a BP Plus device (BP Plus, Uscom, Australia) (Lin *et al.*, 2012).

**FIG. 9.**
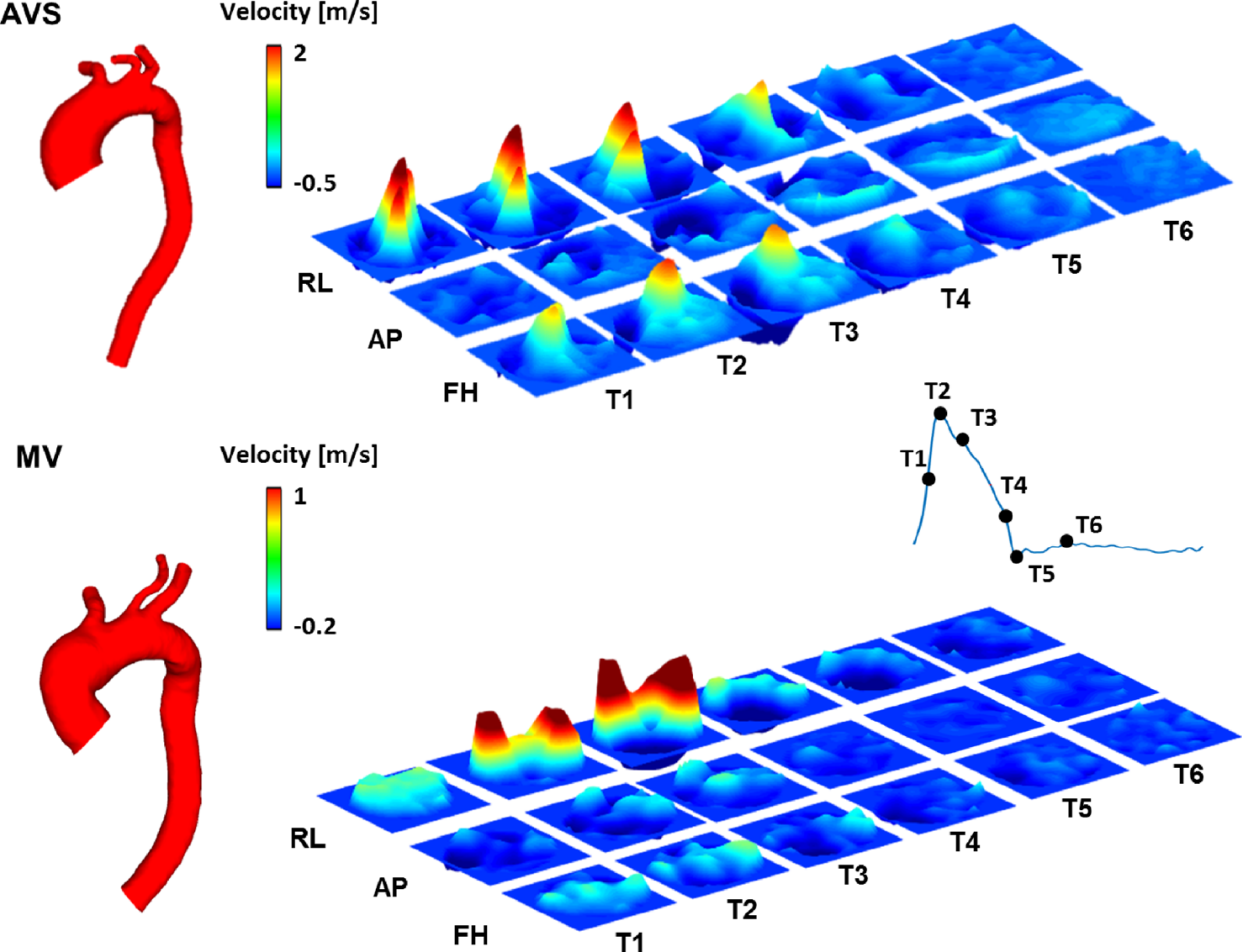
3D velocity profiles extracted from PC-MRI data acquired at the inlet along three directions. T1–T6 correspond to six time points in a cycle. RL, right-left; AP, anterior-posterior; and FH, foot-head.

Patient-specific 3D geometries of the thoracic aorta and aortic branches were reconstructed from MR images by using Mimics (v18.0, Materialise, Leuven, Belgium). An in-house MATLAB (The MathWorks Inc., Natick, MA, United States) tool was used for PC-MR image processing and manual segmentation (Cheng *et al.*, 2016). Figure 9 shows the reconstructed aortic geometry of the two patients along with the spatiotemporal velocity profiles in three orthogonal directions at the ascending aorta inlet. Three structured meshes, consisting of ∼1.6–2.8 × 10^6^ hexahedral elements, were generated using ANSYS ICEM (v15.0, ANSYS Inc., Canonsburg, PA) for both aortic models. These meshes were tested as part of a sensitivity analysis, and the chosen meshes (with ∼2.2 for AVS and ∼2.1 × 10^6^ hexahedral elements for MV) differed from the finer mesh in the predicted maximum and mean WSS by less than 1.5% and 1%, respectively.

The model inlet was just above the aortic root, where the PC-MRI flow mapping planes were located. The acquired images were processed to obtain 3D time-varying velocity profiles for the whole cardiac cycle. The following inlet boundary conditions were investigated in this study:

• 3D velocity profile (3D): the extracted 3D time-varying patient-specific velocity profiles were imposed as inlet BC;
• 1D through-plane velocity profile (TP): only the through-plane velocity component was used as inlet BC. This was obtained from the scalar product of the vector normal to the acquisition plane and the 3D velocity vector obtained from the PC-MRI data;
• Flat profile (FLAT): the extracted velocity information was used to calculate the patient-specific flow waveform, which was imposed with the assumption of a flat profile.

For both 3D and TP inlet BCs, the obtained velocity profiles were mapped on to the 3D global coordinates using a coordinate transfer matrix, which was calculated by using four corresponding reference points between the 2D imaging plane and the 3D coordinates of the model inlet. This allowed for a correspondence to be established between each pixel in the velocity map and a point at the CFD model inlet. Interpolations were then performed to obtain velocities at the mesh nodes and the required computational time points. The details of the method have been described elsewhere (Cheng *et al.*, 2016). For outlet BC at all the model outlets (descending aorta and arch branches), the 3-element Windkessel model was adopted based on the recommendation of a previous study (Pirola *et al.*, 2017). Flow was assumed to be laminar, and blood was considered as a Newtonian fluid, with a constant viscosity of 4 × 10^−3 ^Pa s and a density of 1060 kg m^−3^.

Numerical solutions were obtained using ANSYS CFX (v15.0, ANSYS, Canonsburg, PA, USA). Simulations were performed over several cycles until a periodic solution was reached, and the results obtained in the last cycle were used for the analysis. Velocity data were post-processed in ANSYS CFD-POST (v15.0, ANSYS, Canonsburg, PA, USA) and MATLAB. Time-averaged WSS (TAWSS), oscillatory shear index (OSI), helicity density (*H_k_*), and helicity flow index (HFI) values (Singh *et al.*, 2016a; and Gallo *et al.*, 2012) were calculated using EnSight (v10.1, CEI, Woodlands, TX, USA) and analysed in MATLAB. For the evaluation of HFI, *N_p_* immaterial particles were released from the model inlet at four equally distributed systolic time points.
